# Prognostic Value of Simple Non-Invasive Tests for the Risk Stratification of Incident Hepatocellular Carcinoma in Cirrhotic Individuals with Non-Alcoholic Fatty Liver Disease

**DOI:** 10.3390/cancers15061659

**Published:** 2023-03-08

**Authors:** Angelo Armandi, Gian Paolo Caviglia, Amina Abdulle, Chiara Rosso, Kamela Gjini, Gabriele Castelnuovo, Marta Guariglia, Nuria Perez Diaz del Campo, Daphne D’Amato, Davide Giuseppe Ribaldone, Giorgio Maria Saracco, Elisabetta Bugianesi

**Affiliations:** 1 Department of Medical Sciences, University of Turin, 10126 Turin, Italy; 2 Metabolic Liver Disease Research Program, University Medical Center, Department of Internal Medicine I, Johannes Gutenberg University, 55131 Mainz, Germany

**Keywords:** non-alcoholic fatty liver disease, liver cirrhosis, non-invasive tests, FIB-4 score, hepatocellular carcinoma, metabolic syndrome, BARD score, type 2 diabetes

## Abstract

**Simple Summary:**

In this cohort study of cirrhotic patients with non-alcoholic fatty liver disease, we found that fibrosis-4 score (FIB-4) was associated with incident hepatocellular carcinoma (HCC) over a median follow up of 6 years, independently from metabolic co-factors (obesity and type 2 diabetes). The lowest cut-off of 1.45 to rule out and the highest cut-off of 3.25 to rule in allow for the optimal risk stratification of HCC in this population.

**Abstract:**

Hepatocellular carcinoma (HCC) represents a relevant disease burden in cirrhotic patients with non-alcoholic fatty liver disease (NAFLD). We aimed to investigate the prognostic value of simple non-invasive tests (NITs) (AAR, APRI, BARD, FIB-4) for the stratification of HCC risk development in a cohort of 122 consecutive cirrhotic individuals with NAFLD. Over a median follow up of 5.9 (3.2–9.3) years, 13 (10.7%) developed HCC. Only FIB-4 was associated with HCC risk (HR = 1.27, 95% CI 1.03–1.58, *p* = 0.027). After evaluating different established FIB-4 cut-offs, the lowest cut-off of 1.45 allowed the ruling out of a greater number of patients with a minimal risk of HCC than the 1.3 cut-off (23 vs. 18 patients). Conversely, the cumulative incidence of HCC using the highest cut-off of 3.25 (rule in) was distinctly higher than the 2.67 cut-off (19.4% vs. 13.3%). After multivariate Cox regression analysis, these cut-offs were independently associated with HCC after adjusting for sex, BMI and T2DM (HR = 6.40, 95% CI 1.71–24.00, *p* = 0.006). In conclusion, FIB-4 values of <1.3 and >3.25 could allow for the optimal stratification of long-term HCC risk in cirrhotic individuals with NAFLD.

## 1. Introduction

Non-alcoholic fatty liver disease (NAFLD) represents the most common form of liver disease worldwide, with an estimated prevalence of 25% among the adult population, growing in parallel with the increase in obesity and type 2 diabetes mellitus (T2DM) [1]. NAFLD encompasses different forms of liver injury, from simple intrahepatic steatosis to a progressive form of chronic inflammation (named non-alcoholic steatohepatitis, NASH), which can lead to advanced liver disease, including cirrhosis and its complications [2,3]. In particular, the incidence of hepatocellular carcinoma (HCC) represents a relevant disease burden in this population, with considerable rates of morbidity and mortality [4,5]. In the NAFLD landscape, multiple metabolic risk factors may synergistically promote tumorigenesis through enhanced oxidative stress and cell metabolic pathway derangements [6].

According to the American Association for the Study of the Liver (AASLD) and the European Association for the Study of the Liver (EASL) guidelines, cirrhotic patients should undergo standardized screening modalities for HCC surveillance via six-month abdominal ultrasound [7,8]. However, this approach does not take into account the heterogeneity in cirrhotic individuals, which may imply different degrees of HCC risk according to either clinical features, or the impact of different etiologies as well as the removal of the etiologic agent [9]. In the context of NAFLD, the natural history is highly unpredictable due to multiple environmental factors, hence requiring a more tailored approach for a better risk stratification, with the aim of employing personalized, cost-effective procedures [10].

Non-invasive tests (NITs) have been introduced in clinical practice to overcome the limitation of invasive procedures for diagnostic/stratification purposes (e.g., liver biopsy to assess fibrosis stage or hepatic vein portal gradient (HVPG) to assess portal hypertension). In addition, some NITs have provided strong evidence for prognostication in cirrhotic patients of any etiology at risk for HCC [11]. Most NITs consist of simple scores using readily available clinical–biochemical variables, including fibrosis-4 (FIB-4) score that has been widely validated for the cross-sectional identification of advanced liver fibrosis in the hepatologist referral pathway [12]. In addition, some evidence has been so far provided for a longitudinal assessment of NITs with regard to long-term outcomes in NAFLD [13,14], including liver decompensation and overall mortality. However, less strong evidence exists with regard to HCC incidence in NAFLD using NITs at univocal cut-offs. In one study, only patients with high FIB-4 values, which persisted across repetitive follow up measurements, had a relevant risk of incident HCC [15].

Based on these premises, the aim of the present study was to investigate the prognostic value of simple NITs for the stratification of HCC risk development in NAFLD cirrhotic patients on long-term follow up.

## 2. Materials and Methods

### 2.1. Study Design and Study Population

This retrospective cohort study included consecutive patients with cirrhosis due to NAFLD at their first referral at the outpatient clinic of the Unit of Gastroenterology and Hepatology of A.O.U. Città della Salute e della Scienza di Torino—Molinette Hospital, Turin, Italy, between January 2010 and April 2022.

NAFLD was assessed by either histologic evaluation (macrovescicular steatosis > 5%) or by liver steatosis at abdominal ultrasound, in the absence of other known causes of liver damage (including viral hepatitis, cholesteric/autoimmune liver disease, drug-induced liver injury, use of steatogenic medications) along with the presence of metabolic risk factors (including obesity, T2DM, arterial hypertension, dyslipidemia) [16]. All available clinical, biochemical and anthropometric variables were retrieved at the time of the cirrhosis diagnosis.

Liver stiffness was measured via vibration-controlled transient elastography (VCTE) (FibroScan^®^, Echosens™, Paris, France) in a fasting condition, performed by an expert operator, and using the M or XL probe as appropriate. All measurements were considered technically reliable with an IQR/med ratio of <30%.

**Figure 1:**
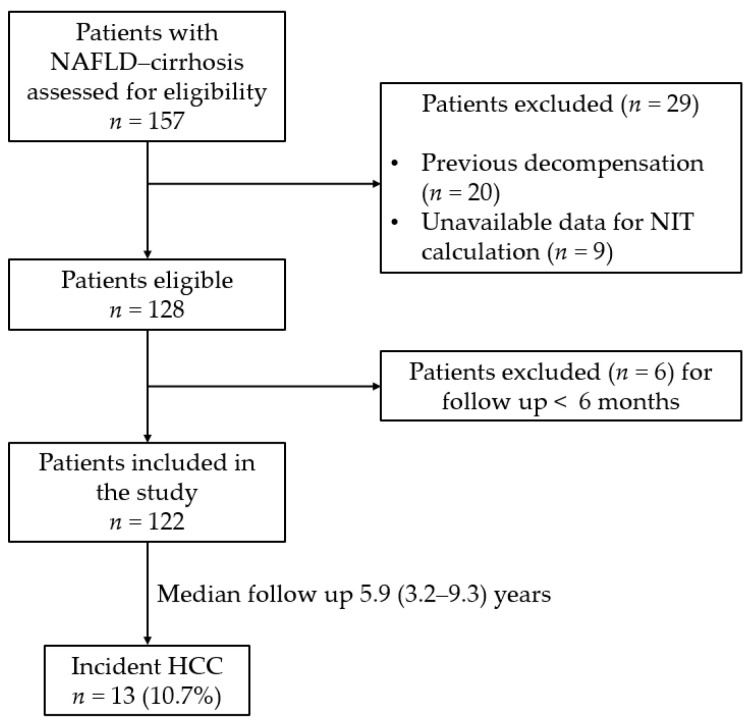
Flow chart of the study. Abbreviations: HCC: hepatocellular carcinoma. NAFLD: non-alcoholic fatty liver disease, NIT: non-invasive test.

A flow chart of study is provided in Figure 1. Inclusion criteria were as follows: aged 18 years or older and a clinical follow up of at least 6 months. Diagnosis of NAFLD cirrhosis was made via histology, or instrumental evidence (including ultrasound or computed tomography imaging), or through indirect signs of portal hypertension (including abdominal collateral circles, platelet count < 150 × 10^9^/L, esophageal varices) [17,18]. Exclusion criteria were as follows: a previous decompensation event (including ascites, gastrointestinal bleeding, hepatic encephalopathy), previous occurrence of HCC, unavailable biochemical data for NIT calculation, and clinical follow up of less than 6 months.

HCC was diagnosed via histologic examination or via suggestive signs at second-level imaging (contrast-enhanced computed tomography or magnetic resonance imaging) according to EASL guidelines [8].

### 2.2. Non-Invasive Tests

The following NITs were calculated at the time of the first referral for each patient according to the original formula:

Aspartate aminotransferase (AST) to alanine aminotransferase (ALT) ratio (AAR) [19]: AST/ALT ratio;

AST to platelets ratio index (APRI) [20]: (AST/AST upper limit normal/Platelets (10^9^/L)) × 100;

Body mass index (BMI), AST/ALT ratio, T2DM score (BARD) [21]: BMI ≥ 28 kg/m^2^ = 1 point, AST/ALT ratio ≥ 0.8 = 2 points, T2DM = 1 point;

FIB-4 [22]: (Age (years) × AST (U/L))/(Platelets (10^9^/L) × √ALT (U/L)).

### 2.3. Statistical Analysis

Continuous variables were reported as median and interquartile ranges (IQR) according to their distribution. Data normality was assessed using the D’Agostino–Pearson test. Categorical variables were reported as number (*n*) and percentage (%). The association between baseline NIT values and HCC occurrence during the follow up was assessed using Cox proportional hazards regression analysis; results were reported as hazard ratio (HR) with the corresponding 95% confidence interval (CI). Survival curves were analyzed with the Kaplan–Meier method; the corresponding *p* values were calculated via a log-rank test. Patients that did not develop HCC were censored at liver transplant or at the last follow up.

A two-tailed *p* value of < 0.05 was considered statistically significant. All the statistical analyses were performed using MedCalc software, v.18.9.1 (MedCalc bvba, Ostend, Belgium).

## 3. Results

### 3.1. Baseline Charcateristics of the Study Cohort

A total of 122 patients with NAFLD cirrhosis were retrospectively included in this study based on the criteria depicted in Figure 1. Demographic, clinical and biochemical characteristics are reported in Table 1. The median age was 62 (51–67) years of age and the male to female ratio was 64/58. A total of 56.6% (*n* = 69) of patients were obese (BMI ≥ 30.0 Kg/m^2^) and 57.4% (*n* = 70) had a diagnosis of T2DM. Liver cirrhosis was detected via liver biopsy in 49 (40.2%) patients; in the remaining 73 (59.8%) patients, cirrhosis diagnosis was achieved from instrumental findings and/or clinical evidence of portal hypertension. Elevated liver enzymes (ALT and/or AST and/or γGT) were found in 105 (86.1%) patients, with ALT > upper limit normal (ULN) in 76 (62.3%) patients, AST > ULN in 37 (30.3%) patients, and γGT > ULN in 96 (78.7%) patients. Liver stiffness was available in nearly the half of the study cohort; in these patients, median stiffness was 20.5 (14.3–27.3) kPa.

### 3.2. Baseline NITs Values and Association with HCC Developemnt

**Figure 2:**
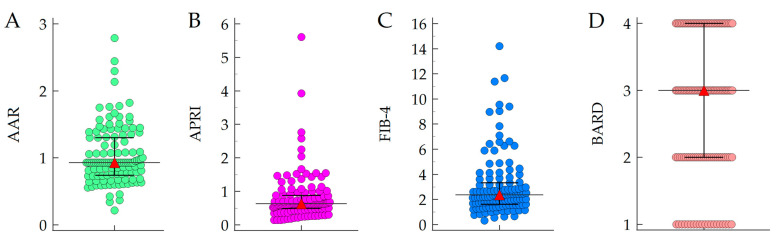
Distribution of NIT values ((**A**): AAR; (**B**): APRI; (**C**): FIB-4; (**D**): BARD) in the study cohort. Dots represent individual values, while red triangles represent median values. Abbreviations: AAR, AST/ALT ratio; APRI, AST to platelet ratio index; FIB-4, fibrosis 4.

Baseline NITs values were calculated for all the 122 patients; median AAR values were 0.93 (0.74–1.30), median APRI values were 0.63 (0.79–0.88), median FIB-4 values were 2.37 (1.63–3.33), and median BARD values were 3 (2–4) (Figure 2).

A total of 122 patients were followed for a median of 5.9 (3.2–9.3) years; during the follow up, 13 (cumulative incidence = 10.7%) patients developed HCC. In our population, the incidence rate was 1.5 per 100 person/year. Among the investigated NITs, only FIB-4 values resulted in a significant association with an increased risk of HCC development during follow up (HR = 1.27, 95% CI 1.03–1.58, *p* = 0.027). No association was observed for AAR (HR = 1.78, 95%CI 0.51–6.31, *p* = 0.369), APRI (HR = 1.50, 95%CI 0.91–2.48, *p* = 0.115) or BARD (HR = 1.29, 95%CI 0.72–2.34, *p* = 0.392).

### 3.3. Stratifcation of the Risk of HCC According to FIB-4

**Figure 3:**
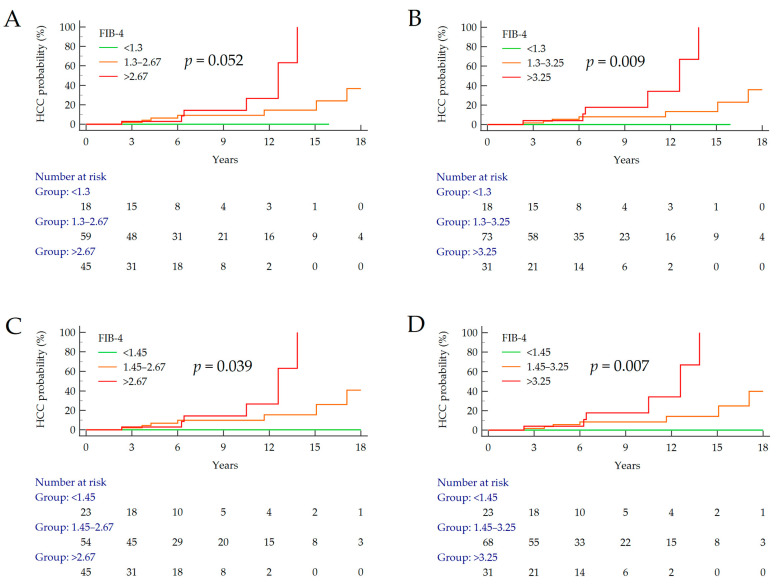
FIB-4 survival curves according to different cut-off combinations ((**A**): 1.3 and 2.67; (**B**): 1.3 and 3.25; (**C**): 1.45 and 2.67; (**D**): 1.45 and 3.25). *p* values were calculated using log-rank test. Abbreviations: FIB-4, fibrosis 4; HCC, hepatocellular carcinoma.

Baseline NITs values were categorized according to the most widely adopted cut-offs in order to investigate which of them were the most appropriate for the stratification of the risk of HCC development in patients with NAFLD cirrhosis [15,22,23]. After Kaplan–Meier analysis, no statistical significance was observed for AAR, APRI and BARD (Supplementary Table S1 and Supplementary Figure S1); only FIB-4 showed different survival curves. Remarkably, no HCC occurred in patients with FIB-4 values below 1.3 and 1.45 (low-risk category); however, a cut-off value of 1.45 allowed us to rule out a greater number of patients with minimal risk of HCC as compared to a cut-off value of 1.3 (23 vs. 18, respectively). Conversely, the cumulative incidence of HCC in patients with FIB-4 of >3.25 (6/31; 19.4%) was distinctly higher compared to patients with FIB-4 of > 2.67 (6/45; 13.3%) (Table 2, Figure 3). According to these findings, a low-risk cut-off of 1.45 and a high-risk cut-off of 3.25 appeared the most appropriate values for the stratification of patients with NAFLD cirrhosis according their individual risk of HCC development during the follow up. Finally, after multivariate Cox regression analysis adjusted for sex, BMI and T2DM, only FIB-4 (<1.45, 1.45–3.25, >3.25) was significantly and independently associated with an increased risk of HCC occurrence (HR = 6.40, 95% CI 1.71–24.00, *p* = 0.006) (Table 3).

## 4. Discussion

In this retrospective cohort study of patients with cirrhosis due to NAFLD, we found that among commonly used NITs, only FIB-4 values were significantly associated with increased incidence of HCC over a median time of 6 years. This significant association persisted after adjusting for major metabolic co-factors including BMI and T2DM. All patients from this cohort had not experienced any liver decompensation event or previous HCC occurrence; hence, this evidence was provided in a homogenous cohort, increasing the plausibility of the findings.

HCC represents one of the most common complications of cirrhosis, leading to relevant morbidity and mortality in this population. In particular, NAFLD offers a favorable background for HCC development, giving the persistent chronic inflammation and enhanced oxidative stress caused by the multiple metabolic-dysfunction-related injuries. In fact, up to one third of NAFLD individuals may develop HCC even in a pre-cirrhotic stage. However, since cirrhosis is the most relevant pre-neoplastic condition for HCC development, we included all consecutive cirrhotic patients at their first referral. A wide validation of non-invasive tools for HCC risk stratification and prognostication is still lacking, and cirrhotic patients are univocally monitored over time following standardized protocols. However, a tailored approach, with the aim of achieving a personalized surveillance strategy would be warranted, for either a cost-effective use of resources or for a better risk-based monitoring over time [24].

Consistent with our findings, some studies have assessed the potential role of non-invasive scores for longitudinal purposes, and FIB-4 showed the best accuracy to predict long-term hard outcomes. In fact, FIB-4 has been associated with liver-related events and overall mortality in NAFLD [13,14,25,26,27,28]. Additionally, one study investigated the FIB-4 changes over time in NAFLD patients, finding that persistently high FIB-4 values were associated with increased incidence of cirrhosis and HCC using a cut-off of 1.45 [15]. Similarly, in our cohort, we found that using the lowest FIB-4 cut-off of 1.45 to rule out HCC allows for a better selection of patients with a minimal risk of developing HCC. In addition, in the present study, the cut-off of 3.25 seemed optimal to identify the highest cumulative incidence of HCC.

In cross-sectional studies, Sterling et al. first assessed the presence of liver fibrosis in HCV/HIV patients using the same cut-offs of 1.45 and 3.25 to rule out and rule in, respectively [22]. On the other hand, another study assessed the risk of advanced fibrosis using 1.30 and 2.67 cut-offs, which did not provide the same evidence in our longitudinal setting (*p* = 0.052) [29]. Consistent with our evidence, a 5-year prospective study on biopsy-proven NAFLD patients assessed a higher risk of death and liver transplantation with FIB-4 of >3.25 (HR = 6.33) [25]. The majority of other studies assessing the risk for long-term outcomes in NAFLD has provided stronger evidence using the 1.30 and 2.67 cut-offs [14,23,26,27,28]. These discrepancies may be partly be explained by the biological variability in the study cohorts and in the different degree of liver disease severity that is translated into the FIB-4 score, with regard to age, portal hypertension (and derivative platelet count) and intrahepatic ongoing inflammation as mirrored by transaminases. In the landscape of NAFLD, where the natural history may be unpredictable, being shaped by multiple environmental factors, a careful stratification of patients is advisable. Based on our findings and in the context of the existing literature, the follow-up evaluation of FIB-4 values would allow for the best characterization of patients into risk categories, which may change over time according to diverse variables [30].

The strength of this study is the well-characterized cohort of NAFLD patients with cirrhosis according to defined criteria and with the long-term follow up that allowed for the longitudinal evaluation. However, some limitations have to be outlined. The retrospective nature of the study did not allow for a complete clinical–biochemical evaluation at the time of diagnosis, leading to a relevant lack of data in some cases (including CHILD class definition and availability of liver stiffness values for the whole cohort) that prevented for an optimal cohort stratification. In addition, the lack of comprehensive baseline biochemical data did not allow for the calculation of other well-established scores and biomarkers (i.e., alpha-fetoprotein (AFP)) in the HCC landscape. Indeed, AFP data availability was even lower (*n* = 27), since the measure of circulating biomarkers is not recommended for the surveillance of patients at risk of HCC in European guidelines [8]. Overall, these limitations are also explained in the view that our aim was to explore the incidence of HCC starting from the cirrhosis diagnosis, which may have occurred prior to our first observation, significantly affecting the availability of the data.

## 5. Conclusions

In conclusion, in this cohort study of NAFLD cirrhotic patients, FIB-4 values are significantly and independently associated with HCC incidence over a median time of 5 years. Among the most validated FIB-4 cut-offs, we found that using the low cut-off of 1.45 (ruling out) and the high cut-off of 3.25 (ruling in) would allow for the optimal risk stratification in this population.

## Figures and Tables

**Table 1:** Baseline characteristics of the study cohort.

Variables	Data
Patients, *n*	122
Age (years), median (IQR) [122]	62 (51–67)
Male sex, *n* (%) [122]	64 (52.5%)
BMI (Kg/m^2^), median (IQR) [122]	30.5 (27.8–33.3)
Lean (BMI < 25.0 Kg/m^2^), *n* (%)	10 (8.2%)
Overweight (BMI 25.0–29.9 Kg/m^2^), *n* (%)	43 (35.2%)
Obese (BMI ≥ 30.0 Kg/m^2^), *n* (%)	69 (56.6%)
T2DM, *n* (%) [122]	70 (57.4%)
Hb (g/dL), median (IQR) [89]	13.9 (12.4–15.1)
WBC count (×10^9^/L), median (IQR) [61]	5.90 (4.77–7.38)
Platelet count (×10^9^/L), median (IQR) [122]	155 (108–198)
ALT (U/L), median (IQR) [122]	42 (24–61)
AST (U/L), median (IQR) [122]	39 (26–47)
γGT (U/L), median (IQR) [122]	97 (45–173)
ALP (U/L), median (IQR) [57]	91 (71–123)
INR, median (IQR) [61]	1.09 (1.01–1.22)
Albumin (g/dL), median (IQR) [54]	4.2 (3.9–4.4)
Total bilirubin (mg/dL), median (IQR) [78]	0.7 (0.5–1.2)
Total cholesterol (mg/dL), median (IQR) [47]	179 (148–205)
HDL cholesterol (mg/dL), median (IQR) [46]	45 (36–59)
Triglycerides (mg/dL), median (IQR) [57]	136 (84–206)
Liver stiffness (kPa), median (IQR) [60]	20.5 (14.3–27.3)

Numbers in brackets indicate the number of patients with available data. Abbreviations: ALP, alkaline phosphatase; ALT, alanine aminotransferase; AST, aspartate aminotransferase; BMI, body mass index; γGT, γ-glutamyl transpeptidase; Hb, hemoglobin; HDL, high-density lipoprotein; INR, international normalized ratio; WBC, white blood cell; T2DM, type 2 diabetes mellitus.

**Table 2:** Cumulative probability and hazard ratios of HCC according to FIB-4 risk category.

FIB-4 Cut-Offs	HCC (*n*)	Cumulative (%)	*p* Value ^1^	HR (95% CI)	*p* Value ^2^
<1.3 1.3–2.67 >2.67	0/18 7/59 6/45	0% 11.9% 13.3%	0.052	3.79 (1.21–1.90)	0.023
<1.3 1.3–3.25 >3.25	0/18 7/73 6/31	0% 9.6% 19.4%	0.009	4.95 (1.57–15.59)	0.006
<1.45 1.45–2.67 >2.67	0/23 7/54 6/45	0% 13.0% 13.3%	0.039	3.86 (1.28–11.66)	0.017
<1.45 1.45–3.25 >3.25	0/23 7/68 6/31	0% 10.3% 19.4%	0.007	4.97 (1.63–15.13)	0.005

^1^ *p* value was calculated via log-rank test. ^2^ *p* value was calculated using Cox regression analysis. Abbreviations: CI, confidence interval; FIB-4, fibrosis 4; HCC, hepatocellular carcinoma; HR, hazard ratio; *n*, number.

**Table 3:** Multivariate adjusted Cox regression analysis for baseline factors associated with HCC development. Hazard ratios and 95% CIs of outcome by risk score category.

Baseline Variables	HR (95% CI)	*p* Value
FIB-4 (<1.45, 1.45–3.25, >3.25)	6.40 (1.71–24.00)	0.006
Male sex	0.75 (0.32–4.86)	0.748
BMI (Kg/m^2^)	0.94 (0.82–1.08)	0.398
T2DM	1.06 (0.27–4.13)	0.938

Abbreviations: BMI, body mass index; CI, confidence interval; FIB-4, fibrosis 4; HR, hazard ratio, T2DM, type 2 diabetes mellitus.

## Data Availability

The data presented in this study are available on request from the corresponding author.

## Supplementary Materials

The following supporting information can be downloaded at: https://www.mdpi.com/article/10.3390/cancers15061659/s1, Table S1. Cumulative probability and hazard ratios of HCC by AAR, APRI, and BARD risk category; Figure S1. Survival curves of AAR (A), APRI (B) and BARD (C). AAR values were categorized according to tertiles distribution. p values were calculated by log-rank test. Abbreviations: AAR, AST to ALT ratio; APRI, AST to platelets ratio index; BARD, body mass index, AST/ALT ratio, diabetes mellitus score; HCC, hepatocellular carcinoma.

## References

[1] Younossi Z.M., Koenig A.B., Abdelatif D., Fazel Y., Henry L., Wymer M. (2016). Global epidemiology of nonalcoholic fatty liver disease-Meta-analytic assessment of prevalence, incidence, and outcomes. Hepatology.

[2] Bugianesi E., Leone N., Vanni E., Marchesini G., Brunello F., Carucci P., Musso A., Paolis P.D., Capussotti L., Salizzoni M. (2002). Expanding the natural history of nonalcoholic steatohepatitis: From cryptogenic cirrhosis to hepatocellular carcinoma. Gastroenterology.

[3] Armandi A., Bugianesi E. (2021). Natural history of NASH. Liver Int..

[4] Reddy S.K., Steel J.L., Chen H.W., DeMateo D.J., Cardinal J., Behari J., Humar A., Marsh J.W., Geller D.A., Tsung A. (2012). Outcomes of curative treatment for hepatocellular cancer in nonalcoholic steatohepatitis versus hepatitis C and alcoholic liver disease. Hepatology.

[5] Huang D.Q., El-Serag H.B., Loomba R. (2021). Global epidemiology of NAFLD-related HCC: Trends, predictions, risk factors and prevention. Nat. Rev. Gastroenterol. Hepatol..

[6] Marengo A., Rosso C., Bugianesi E. (2016). Liver Cancer: Connections with Obesity, Fatty Liver, and Cirrhosis. Annu. Rev. Med..

[7] Marrero J.A., Kulik L.M., Sirlin C.B., Zhu A.X., Finn R.S., Abecassis M.M., Roberts L.R., Heimbach J.K. (2018). Diagnosis, Staging, and Management of Hepatocellular Carcinoma: 2018 Practice Guidance by the American Association for the Study of Liver Diseases. Hepatology.

[8] European Association for the Study of the Liver (2018). EASL Clinical Practice Guidelines: Management of hepatocellular carcinoma. J. Hepatol..

[9] Ciancio A., Ribaldone D.G., Spertino M., Risso A., Ferrarotti D., Caviglia G.P., Carucci P., Gaia S., Rolle E., Sacco M. (2023). Who Should Not Be Surveilled for HCC Development after Successful Therapy with DAAS in Advanced Chronic Hepatitis C? Results of a Long-Term Prospective Study. Biomedicines.

[10] Singal A.G., Lok A.S., Feng Z., Kanwal F., Parikh N.D. (2022). Conceptual Model for the Hepatocellular Carcinoma Screening Continuum: Current Status and Research Agenda. Clin. Gastroenterol. Hepatol..

[11] Caviglia G.P., Troshina G., Santaniello U., Rosati G., Bombaci F., Birolo G., Nicolosi A., Saracco G.M., Ciancio A. (2022). Long-Term Hepatocellular Carcinoma Development and Predictive Ability of Non-Invasive Scoring Systems in Patients with HCV-Related Cirrhosis Treated with Direct-Acting Antivirals. Cancers.

[12] European Association for the Study of the Liver (2021). EASL Clinical Practice Guidelines on non-invasive tests for evaluation of liver disease severity and prognosis-2021 update. J. Hepatol..

[13] Younes R., Caviglia G.P., Govaere O., Rosso C., Armandi A., Sanavia T., Pennisi G., Liguori A., Francione P., Gallego-Duran R. (2021). Long-term outcomes and predictive ability of non-invasive scoring systems in patients with non-alcoholic fatty liver disease. J. Hepatol..

[14] Hagström H., Nasr P., Ekstedt M., Stål P., Hultcrantz R., Kechagias S. (2019). Accuracy of Noninvasive Scoring Systems in Assessing Risk of Death and Liver-Related Endpoints in Patients With Nonalcoholic Fatty Liver Disease. Clin. Gastroenterol. Hepatol..

[15] Cholankeril G., Kramer J.R., Chu J., Yu X., Balakrishnan M., Li L., El-Serag H.B., Kanwal F. (2022). Longitudinal changes in fibrosis markers are associated with risk of cirrhosis and hepatocellular carcinoma in non-alcoholic fatty liver disease. J. Hepatol..

[16] Boyle M., Masson S., Anstee Q.M. (2018). The bidirectional impacts of alcohol consumption and the metabolic syndrome: Cofactors for progressive fatty liver disease. J. Hepatol..

[17] Gaia S., Campion D., Evangelista A., Spandre M., Cosso L., Brunello F., Ciccone G., Bugianesi E., Rizzeto M. (2015). Non-invasive score system for fibrosis in chronic hepatitis: Proposal for a model based on biochemical, FibroScan and ultrasound data. Liver Int..

[18] Caviglia G.P., Armandi A., Rosso C., Gaia S., Aneli S., Rolle E., Abate M.L., Olivero A., Nicolosi A., Guariglia M. (2021). Biomarkers of Oncogenesis, Adipose Tissue Dysfunction and Systemic Inflammation for the Detection of Hepatocellular Carcinoma in Patients with Nonalcoholic Fatty Liver Disease. Cancers.

[19] De Ritis F., Coltorti M., Giusti G. (1957). An enzymic test for the diagnosis of viral hepatitis; the transaminase serum activities. Clin. Chim. Acta..

[20] Wai C.T., Greenson J.K., Fontana R.J., Kalbfleisch J.D., Marrero J.A., Conjeevaram H.S., Lok A.S. (2003). A simple noninvasive index can predict both significant fibrosis and cirrhosis in patients with chronic hepatitis C. Hepatology.

[21] Harrison S.A., Oliver D., Arnold H.L., Gogia S., Neuschwander-Tetri B.A. (2008). Development and validation of a simple NAFLD clinical scoring system for identifying patients without advanced disease. Gut.

[22] Sterling R.K., Lissen E., Clumeck N., Sola R., Correa M.C., Montaner J., Sulkowski M.S., Torriani F.J., Dieterich D.T., Thomas D.L. (2006). Development of a simple noninvasive index to predict significant fibrosis in patients with HIV/HCV coinfection. Hepatology.

[23] Angulo P., Bugianesi E., Bjornsson E.S., Charatcharoenwitthaya P., Mills P.R., Barrera F., Haflidadottir S., Day C.P., George J. (2013). Simple noninvasive systems predict long-term outcomes of patients with nonalcoholic fatty liver disease. Gastroenterology.

[24] Ioannou G.N. (2021). HCC surveillance after SVR in patients with F3/F4 fibrosis. J. Hepatol..

[25] Sebastiani G., Alshaalan R., Wong P., Rubino M., Salman A., Metrakos P., Deschenes M., Ghali P. (2015). Prognostic Value of Non-Invasive Fibrosis and Steatosis Tools, Hepatic Venous Pressure Gradient (HVPG) and Histology in Nonalcoholic Steatohepatitis. PLoS ONE.

[26] Kim D., Kim W.R., Kim H.J., Therneau T.M. (2013). Association between noninvasive fibrosis markers and mortality among adults with nonalcoholic fatty liver disease in the United States. Hepatology.

[27] Vieira Barbosa J., Milligan S., Frick A., Broestl J., Younossi Z., Afdhal N.H., Lai M. (2022). Fibrosis-4 Index as an Independent Predictor of Mortality and Liver-Related Outcomes in NAFLD. Hepatol. Commun..

[28] Önnerhag K., Hartman H., Nilsson P.M., Lindgren S. (2019). Non-invasive fibrosis scoring systems can predict future metabolic complications and overall mortality in non-alcoholic fatty liver disease (NAFLD). Scand. J. Gastroenterol..

[29] Shah A.G., Lydecker A., Murray K., Tetri B.N., Contos M.J., Sanyal A.J., Network N.C.R. (2009). Comparison of noninvasive markers of fibrosis in patients with nonalcoholic fatty liver disease. Clin. Gastroenterol. Hepatol..

[30] Ishiba H., Sumida Y., Tanaka S., Yoneda M., Hyogo H., Ono M., Fujii H., Eguchi Y., Suzuki Y., Yoneda M. (2018). The novel cutoff points for the FIB4 index categorized by age increase the diagnostic accuracy in NAFLD: A multi-center study. J. Gastroenterol..

