# Supplementary material for: Prognostic Value of Simple Non-Invasive Tests for the Risk Stratification of Incident Hepatocellular Carcinoma in Cirrhotic Individuals with Non-Alcoholic Fatty Liver Disease
Source: Cancers (Basel). 2023 Mar 8;15(6):1659. doi: 10.3390/cancers15061659 (PMC10046647; doi:10.3390/cancers15061659)
Supplement: Supplementary file 1 [file cancers-15-01659-s001.zip › cancers-2218100-supplementary.pdf]

cancers

MDPI

Supplementary Material

Prognostic Value of Simple Non-Invasive Tests for the Risk Stratification of Incident Hepatocellular Carcinoma in Cirrhotic Individuals with Non-Alcoholic Fatty Liver Disease

Angelo Armandi [1,2,†], Gian Paolo Caviglia [1,†], Amina Abdulle [1], Chiara Rosso [1], Kamela Gjini [1], Gabriele Castelnuovo [1], Marta Guariglia [1], Nuria Perez Diaz del Campo [1], Daphne D'Amato [1], Davide Giuseppe Ribaldone [1], Giorgio Maria Saracco [1] and Elisabetta Bugianesi [1,*]

1 Department of Medical Sciences, University of Turin, 10126 Turin, Italy
2 Metabolic Liver Disease Research Program, University Medical Center, Department of Internal Medicine I, Johannes Gutenberg University, 55131 Mainz, Germany
* Correspondence: elisabetta.bugianesi@unito.it
† These authors equally contributed to the manuscript.

Table S1. Cumulative probability and hazard ratios of HCC by AAR, APRI, and BARD risk category.

NITs	HCC (*n*)	Cumulative (%)	*p* value[1]	HR (95% CI)	*p* value[2]
AAR T1	2/41	4.9%			
AAR T2	6/40	15.0%	0.204	2.03 (0.87–4.71)	0.204
AAR T3	5/41	12.1%			
APRI <0.5	1/32	3.1%			
APRI 0.5–1.5	10/79	12.7%	0.062	3.41 (0.89–13.03)	0.073
APRI >1.5	2/11	18.2%			
BARD 0–1	1/20	5.0%			
BARD 2–3	8/66	12.1%	0.820	1.19 (0.49–2.86)	0.703
BARD 4	4/36	11.1%			

[1] *p* value was calculated by log-rank test. [2] *p* value was calculated by Cox regression analysis. Abbreviations: AAR, AST to ALT ratio; APRI, AST to platelets ratio index; BARD, body mass index, AST/ALT ratio, diabetes mellitus score; CI, confidence interval; HCC, hepatocellular carcinoma; HR, hazard ratio; *n*, number.

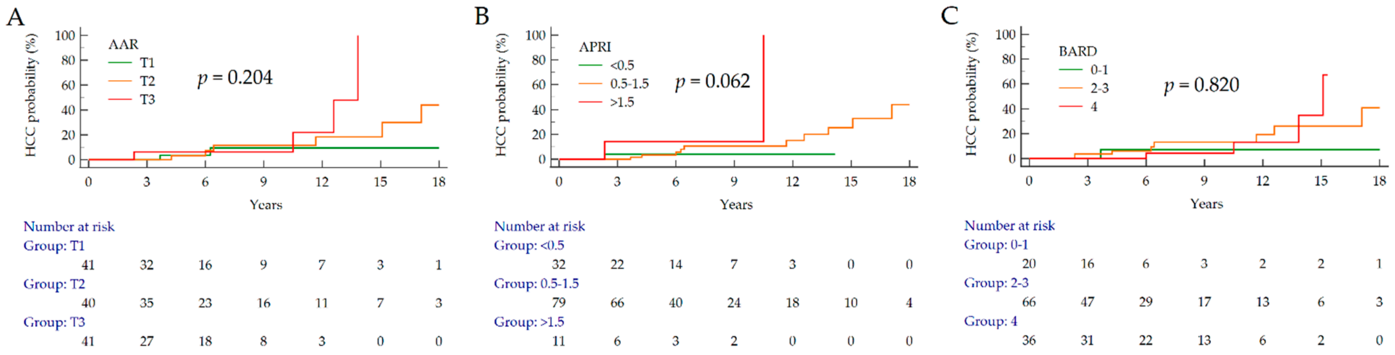

Figure S1. Survival curves of AAR (A), APRI (B) and BARD (C). AAR values were categorized according to tertiles distribution. *p* values were calculated by log-rank test. Abbreviations: AAR, AST to ALT ratio; APRI, AST to platelets ratio index; BARD, body mass index, AST/ALT ratio, diabetes mellitus score; HCC, hepatocellular carcinoma.
